# Preparation of Pd supported on La(Sr)-Mn-O Perovskite by microwave Irradiation Method and Its Catalytic Performances for the Methane Combustion

**DOI:** 10.1038/srep19511

**Published:** 2016-01-19

**Authors:** Wei Wang, Fulong Yuan, Xiaoyu Niu, Yujun Zhu

**Affiliations:** 1 Key Laboratory of Functional Inorganic Material Chemistry (Heilongjiang University), Ministry of Education, School of Chemistry and Materials, Heilongjiang University, Harbin, 150080 P. R. China

## Abstract

In this work, a series of palladium supported on the La_0.8_Sr_0.2_MnO_3.15_ perovskite catalysts (Pd/LSM-x) with different Pd loading were prepared by microwave irradiation processing plus incipient wetness impregnation method and characterized by XRD, TEM, H_2_-TPR and XPS. These catalysts were evaluated on the lean CH_4_ combustion. The results show that the Pd/LSM-x samples prepared by microwave irradiation processing possess relative higher surface areas than LSM catalyst. The addition of Pd to the LSM leads to the increase in the oxygen vacancy content and the enhancement in the mobility of lattice oxygen which play an important role on the methane combustion. The Pd/LSM-3 catalysts with 4.2wt% Pd loading exhibited the best performance for CH_4_ combustion that temperature for 10% and 90% of CH_4_ conversion is 315 and 520 °C.

Natural gas (>90% methane, CH_4_) is promising alternative sources for heat and energy production^1^. The cleaner emission of natural gas engines, i.e., less NOx, SOx, and particulates compared to diesel engines, is also the merit of natural gas^2^,^3^. They are widely used for power generation in gas turbines and natural gas vehicles have become an increasingly important. However, the emission of CH_4_ should be strictly controlled because methane has around 20 times higher greenhouse effect than CO_2_^4^. Therefore, it is important to totally oxidize methane and remove as much of the unburned hydrocarbon as possible prior to its release into the atmosphere. The conventional thermal combustion of methane requires very high temperature (up to 1600 °C), resulting in production of noxious by-products such as NOx^5^. The catalytic combustion has been considered as an effective measure to dispose lean methane for pollution abatement as well as energy utilization at low temperature^6^,^7^,^8^,^9^.

Perovskite (ABO_3_) is a versatile catalyst which has been used in methane partial oxidation^10^. This can be attributed to its unique crystalline structure, ion mobility, and thermal stability^10^,^11^,^12^. It was generally reported that, among the possible compositions, cobalt and manganese-based solids exhibited much higher activities in B position in the structure^13^,^14^,^15^. Levasseur and Kaliaguine^16^ prepared a series of LaBO_3_ (B = Co, Mn and Fe) catalysts by adopting an approach called the “reactive grinding method”, and found that the catalytic performance for methanol oxidation followed the sequence LaMnO_3_ > LaCoO_3_ > LaFeO_3_. Most of the studies on perovskite are based on lanthanum placed in A-position^17^. It has been proven that among the ABO_3_, the La_1-x_Sr_x_MO_3_ (M = Co, Mn; x = 0–0.4) materials show the excellent catalytic performance for the methane combustion^18^,^19^,^20^.

Palladium-based catalysts are currently recognized as the most active in the total combustion of methane and other hydrocarbons, and boast the largest use in the catalytic cleanup of emissions from stationary sources under lean-burn conditions at low temperatures^21^,^22^. As has been found in several studies, there is a number of factors influencing the Pd catalytic activity, the type of palladium precursors^23^, the state of the active Pd component (the nature of the active sites)^24^, and the nature of the support^25^,^26^,^27^. In particular, it is known that the oxidation state of Pd is very important for catalytic methane combustion. Diannan *et al.*^28^ suggested that the active phase of the Pd/Al_2_O_3_ catalyst is PdO or mixed Pd^0^/PdO. R.J. Farrauto *et al.*^29^ considered that at least two distinct oxygen/palladium species are present in palladium oxide supported on alumina decomposes. Once the temperature of the catalyst exceeds 800 °C and palladium metal is formed it does not re-oxidize until the temperature falls to about 650 °C. In fact, it can be confirmed that the existence of a threshold temperature level for the catalyst activation corresponds to the temperature required for the decomposition of Pd oxide species and consequent segregation of metallic Pd onto the surface of the catalyst^30^.

Activity and stability of a catalyst are strongly related to its properties, which are determined by the preparation method adopted. The traditional methods e.g., sol-gel^31^, citrate^32^, solid-state reaction^33^ and co-precipitation^34^, involve in high-temperature and long time. This could damage to the environment and waste materials. In the late 1980 s, microwave assisted synthesis was introduced into the field of materials^35^. It has been reported that the perovskite of uniform particle size and a better oxidation activity can be attained in microwave route^36^. In our previous work, we had investigated the preparation of La-Mn-O perovskite catalyst by microwave irradiation method (MIM) and sol-gel method, and found that the La-Mn-O perovskite catalyst prepared by microwave method showed much better performance for methane combustion^37^. In this study, we investigated the preparation and characterization of La_0.8_Sr_0.2_MnO_3.15_ (LSM) perovskite catalyst by microwave irradiation method and its application used as support to obtained Pd/LSM catalysts by combining the incipient wetness impregnation and microwave irradiation method. The catalytic performances of the Pd/LSM catalysts and the role of Pd species were studied for methane combustion.

## Results and Discussion

### XRD characterization

**Figure 1:**
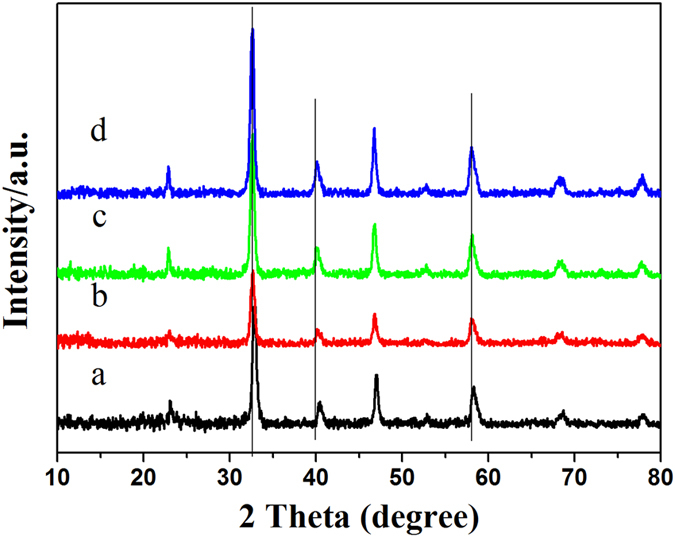
XRD patterns of LSM and Pd/LSM-x catalysts (**a**) LSM, (**b**) Pd/LSM-1, (**c**) Pd/LSM-2, (**d**) Pd/LSM-3.

The LSM and Pd/LSM-x (x = 1, 2 and 3) catalysts with different Pd loadings were prepared by microwave irradiation method. The XRD patterns of the LSM and Pd/LSM-X catalysts are shown in Fig. 1. All of the Bragg diffraction peaks in the *2θ* range of 10–80 ° could be well indexed. For the signal of LSM intensive and sharp diffraction peaks at *2θ* values of 23.07, 32.85, 40.35, 46.97, 52.72, 58.20, 68.64 and 77.81 ° observed here can be primarily attributed to LaMnO_3.15_ perovskite-type oxides (JCPDS 50–0298). The diffraction peaks of Pd/LSM-x located at about *2θ* values of 22.93, 32.58, 40.13, 46.75, 52.32, 58.07, 68.11 and 77.67 ° shift slightly toward lower *2θ* degree compared with the LSM. Table S1 lists the *2θ* values of the diffraction peaks for the LSM and Pd/LSM-x catalysts. It is nearly identical to their corresponding perovskite La_0.8_Sr_0.2_MnO_3_ structures (JCPDS 53–0058), indicating part of the oxygen was reduced under the presence of ethylene glycol (EG). It could be assigned to the removal of the nonstoichiometric excess oxygen in the prepared process by MIM using EG. For the patterns of the Pd/LSM-x catalysts, no obvious diffraction peaks of PdO or Pd were observed, suggesting the high dispersion of palladium with the low Pd loading. The XRD results can clearly confirm that the way of microwave synthesis used in this work was sufficient to crystallize the precursor into the perovskite structure during a very short time in the synthesis step. In addition, the intensities of diffraction peaks increase with the addition of Pd amount, suggesting the increase in the crytallinity. However, the average particle sizes calculated from XRD patterns based on Scherrer’s equation are 15.0, 15.8, 16.0 and 16.1 nm for LSM Pd/LSM-1, Pd/LSM-2 and Pd/LSM-3, respectively, which indicates that the average particle sizes increase slightly with the addition of Pd.

The Pd content is 1.2%, 2.6% and 4.2% for the Pd/LSM-1 Pd/LSM-2 and Pd/LSM-3 by ICP-AES analysis, respectively (Table 1). The BET surface areas of the LSM and Pd/LSM-x catalysts are in the range of 15–41 m^2^/g with different error shown in Table 1. Compared with the LSM, it’s noted that the supporting Pd on the LSM perovskite oxides has a positive effect on surface areas. The BET surface area is 15 m^2^·g^−1^ for the LSM, while it increased from 19 m^2^·g^−1^ to 41 m^2^·g^−1^ with the Pd amount for the Pd/LSM-x catalysts, which indicates MIM using EG may induce surface reconstruction that would be able to enhance the specific surface area by the interactions between the Pd and LSM.

### TEM characterization

**Figure 2:**
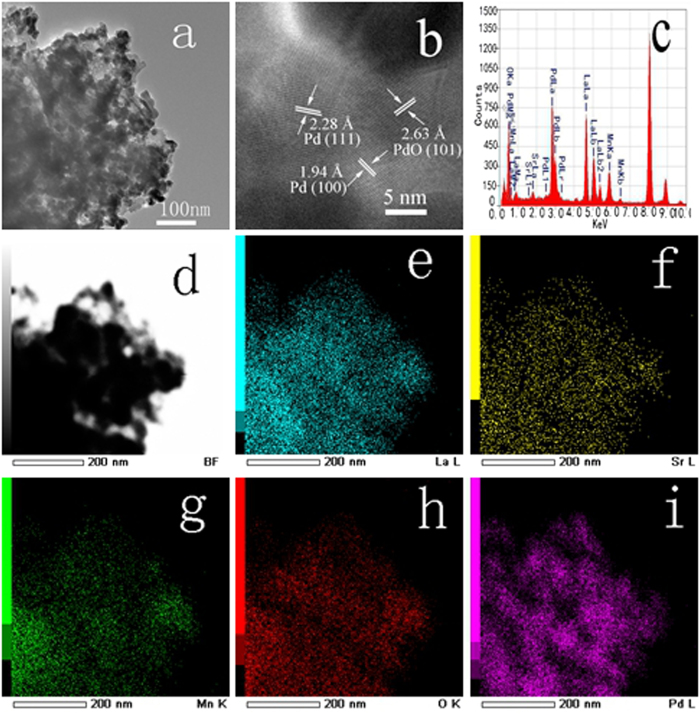
TEM images (**a,b**) at increasing magnifications of Pd/LSM-2 sample. Also shown are EDX mapping results (**d–i**) of Pd/LSM-2.

The TEM and elemental micrograph were conducted to investigate the morphology of the Pd/LSM-x using the Pd/LSM-2 as sample displayed in Fig. 2. From Fig. 2a, it is difficult to distinguish the Pd and LSM phase in the low resolution TEM, which may be attributed to their similar contrast in the TEM image and Pd good dispersion on the LSM. In the HRTEM of the Pd/LSM-2 sample given in Fig. 2b, there were clear lattice fringes, revealed that Pd nanoparticles in the catalyst were bound with (100) facets, and (111) facets, and the (100) and (111) fringes in the spherical nanoparticles exhibited periods of 1.94 and 2.28 Å, respectively, as expected for the lattice spacing of face-centered cubic(fcc) Pd^38^. Meanwhile, it is found that the lattice spacings of nanoparticle is 2.63 Å, which are indexed as the (101) plane of tetragonal PdO^39^. The EDX spectrum of the Pd/LSM-2 (Fig. 2c) exhibits the presence of strong signals of La, Sr, Mn, O and Pd elements. The mapping results of the Fig. 2d zone are presented in Fig. 2e–i in which the distributions of La, Sr, Mn, O and Pd elements are the very same. It indicates that the Pd element is evenly dispersed among La, Sr, Mn and O elements. Figure S1 show the results of TEM for the Pd/LSM-1 and Pd/LSM-3, which is similar to that of Pd/LSM-2. Thus, it is difficult to measure accurately the Pd particle size and their distributions based on the above reasons. The particle size Pd species was evaluated in range of 8–20 nm and the mean particle size is about 12 ± 2 nm based the TEM images. TEM results show that the Pd supported on LSM catalysts were successfully synthesized by microwave method, and in which there are metallic Pd and PdO phase.

### H_2_-TPR characterization

**Figure 3:**
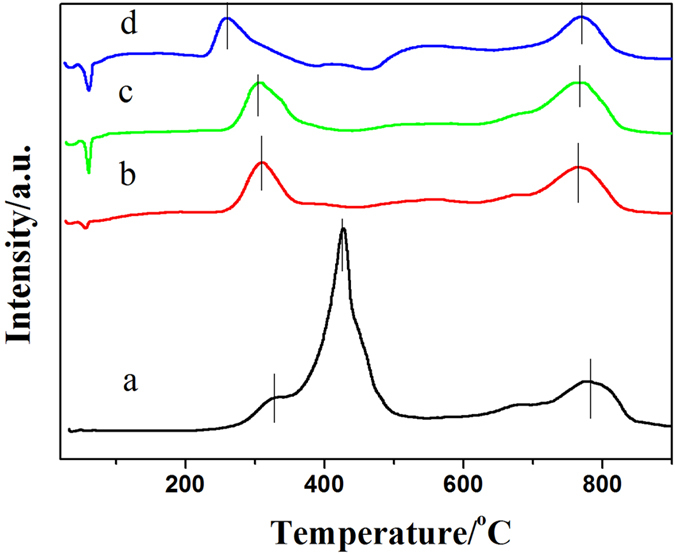
H_2_-TPR profiles of (**a**) LSM, (**b**) Pd/LSM-1, (**c**) Pd/LSM-2, (**d**) Pd/LSM-3.

To investigate the reducibility of the LSM and Pd/LSM-x catalysts, H_2_-TPR was performed and shown in Fig. 3. As is evident from the results in Fig. 3 and a quantitative analysis is summarized in Table 1. The LSM catalyst shows three hydrogen uptake peaks. The first peak shows the hydrogen uptake between 270 and 360 °C with the peak presented at 333 °C. According to the literature^37^, the first peak for the LSM is attributed to the reduction of a fraction of oxygen adsorbed on the surface of the catalysts. The second peak at 426 °C can be seen obviously in the H_2_-TPR curve for the LSM catalyst. To obtain the detailed information about the reduction process, the LSM sample after reduction at 500 °C was characterized by XRD. According to the results shown in Figure S2, the LSM sample after reduction became a phase of LaMnO_3_ perovskite oxide. Thus, the reduction peak at 427 °C for LSM should be assigned to the reduction of Mn^4+^ to Mn^3+^ accompanying the reduction of the nonstoichiometric oxygen^12^,^22^,^37^. The third reduction peak of the LSM sample is in a wide range from 710 °C to 850 °C, which is ascribed to the reduction of Mn^3+^ to Mn^2+^ 12,22,37,40. The Pd/LSM-x catalysts show only one wide peak in the low temperature region between 209 and 470 °C, which can be attributable to the reduction of oxygen adsorbed on the catalysts surface and a few amount of Mn^4+^. Similar to LSM sample, the Pd/LSM-x sample also shows a main reduction peak from 700 °C to 870 °C, which can also be attributable to the reduction of lattice oxygen corresponding the reduction of Mn^3+^. Moreover, each of the Pd/LSM-x samples at below 100 °C has a negative peak, indicating the presence of metal Pd. In H_2_-TPR, metal Pd can absorb a certain mount of hydrogen at low temperature, and then the adsorbed H_2_ will be released with the increase in the temperature. It is noticed that the released H_2_ amount increases with the Pd loading shown in Table 1. The reductive temperature over the Pd/LSM-x catalysts and the H_2_ consumption amount of the referred peak were also gathered in Table 1. The reduced temperature for the low temperature reduced peak is 326, 309, 305 and 261 °C for the LSM, Pd/LSM-1, Pd/LSM-2 and Pd/LSM-3 catalysts, respectively. From the above results, it is clearly seen that the adsorbed oxygen of the Pd/LSM-x samples can be reduced at a much lower temperature than that of the LSM sample, meanwhile, the reduced temperature decreases with the increase in the Pd loading amount. It could be explained that the reduction of the support is advantage by the H_2_-spillover from metallic Pd. In addition, the H_2_ consumption amount of the first reduced peak increase from 0.36, 0.43, 0.46 mmol/g to 0.50 mmol/g for LSM, Pd/LSM-1, Pd/LSM-2 and Pd/LSM-3 catalysts, suggesting the increase in the number of oxygen vacancy with the Pd loading. Compared with the LSM, the reduced temperature for lattice oxygen also shift from 782 °C to 766 °C for the Pd/LSM samples, which indicates the mobility of lattice oxygen is enhanced for the Pd/LSM samples.

### O_2_-TPD characterization

**Figure 4:**
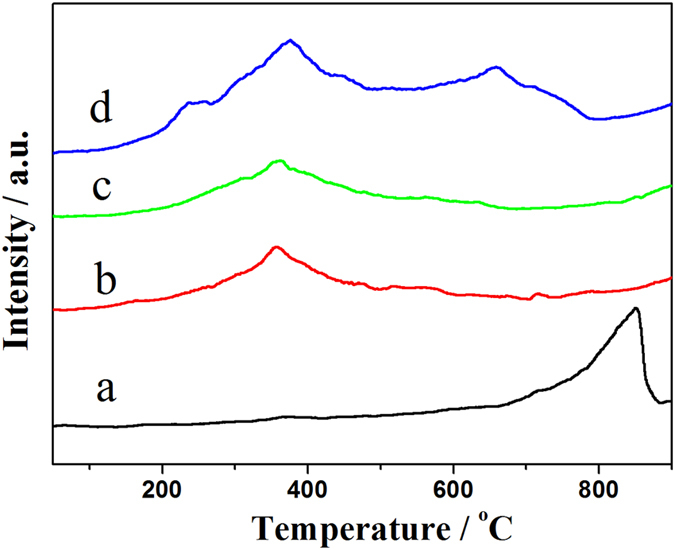
O_2_-TPD profiles of (**a**) LSM, (**b**) Pd/LSM-1, (**c**) Pd/LSM-2, (**d**) Pd/LSM-3.

Figure 4 shows the O_2_-TPD profiles of the LSM and Pd/LSM-x catalysts. In the O_2_-TPD profiles of the LSM, there are two desorption peaks with a weak at low temperature and a strong at high temperature. The weak low temperature peak at 375 °C is assigned to the desorption of the adsorbed oxygen on the oxygen vacancies, and the high temperature peak at 848 °C is attributed to the desorption of the lattice oxygen^12^,^22^. Compared with the LSM, the oxygen desorption show a wide peak from 100 °C to 800 °C for each of Pd/LSM samples. The low-temperature peak in the temperature range of 50–450 °C is related to desorption of adsorbed oxygen on the surface of the catalysts. These results indicate that oxygen vacancies exist on the surface of both Pd/LSM-x and LSM. Pd/LSM-3 and LSM samples in the temperature of 550–900 °C, it is ascribed to the oxygen from crystal lattice of non-stoichiometric oxygen and reduction of manganese^12^,^22^. The Pd/LSM-x catalysts show a broad O_2_ desorption peak in the temperature range of 50–500 °C, and Pd/LSM-3 sample the desorption temperature for lattice oxygen also shift from 848 °C to 659 °C compared with the LSM. It can be deduced that the mobility of lattice oxygen was increased. It is quite consistent with the results of the TPR.

### XPS characterization

**Figure 5:**
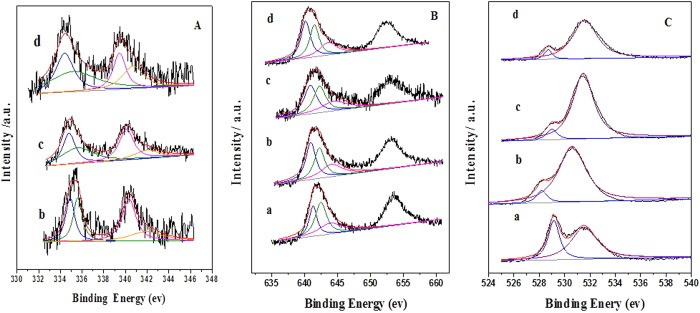
XPS spectra and curve-fitting for LSM and Pd/LSM-x catalysts (**A**) Pd3d_5/2_; (**B**) Mn2p_3/2_; (**C**) O1s; (**a**) LSM; (**b**) Pd/LSM-1; (**c**) Pd/LSM-2; (**d**) Pd/LSM-3.

XPS is an efficient technique for probing the surface element compositions and surface species of a catalyst. Figure 5A presents the results of curve-fitting for Pd 3d_5/2_ spectra. The binding energy at 334.4–334.9 eV could be assigned to Pd^0^ species. The binding energy located from 335.2 to 335.6 eV represents the fraction of Pd in the +2 oxidation state. It is noteworthy that the binding energy shifts to low value with the increase in the Pd loading. Such phenomenon is likely to be due to an interaction between Pd and the LSM perovskite. For the sake of clarity, the surface concentrations of both Pd^2+^ and Pd^0^ species are provided as the ratio of Pd^2+^ to Pd^0^ (Pd^2+^/Pd^0^). Table 2 reports the XPS results in terms of relative concentrations of main surface elements. The value of Pd^2+^/Pd^0^ decreases from 1.4 for Pd/LSM-3 to 1.0 for Pd/LSM-1. It is proportionally more pronounced in the case of Pd/LSM-1 catalyst with lower Pd loading than Pd/LSM-3 sample. Pd is mainly present in all Pd/LSM-x samples as ionic Pd^0^ and a fraction of Pd^2+^.

The Mn2p spectrum reveals three components with binding energy of the Mn2p_3/2_ electrons shown in Fig. 5B. The binding energies in the range of 641.5–642.5 and 640.2–641.3 eV are attributed to the surface Mn^4+^ and Mn^3+^ species, respectively, and the binding energy at 643.6–644.5 eV belongs to the satellite peak^37^. The amount of Mn^4+^ and Mn^3+^ species was analyzed wit the deconvolution obtained by fitting Gaussian peaks after Shirley-ackground subtraction (Table 2). The ratio of Mn^4+^ to Mn^3+^ species is 1.6 for the LSM sample, however, it decreases to 1.1, 0.91 and 0.87 for the Pd/LSM-1, Pd/LSM-2 and Pd/LSM-3 samples, respectively. It is clear that the amount of Mn^4+^ on the surface of the Pd/LSM-x samples is lower than that of the LSM sample, suggesting the Mn^4+^ is reduced partly by ethylene glycol in the synthesis process with the addition of Pd. This phenomenon is consistent with the results of the H_2_-TPR and XRD results.

Figure 5C gives the O1s spectra of the LSM and Pd/LSM-x catalysts. In the XPS spectrum of O1s, the peaks located at about 531 and 529 eV are attributed to adsorbed oxygen on the oxygen vacancy (O_ads_) and lattice oxygen (O_lat_), respectively^40^. The variations in the amount of O_ads_ and O_lat_ for the O1s peak were observed. The ratio of O_ads_ to O_lat_ on the surface of the LSM sample is 1.9, however, it is much higher six times for the Pd/LSM-x samples than that of the LSM sample. Moreover, the surface O_ads_ species concentration (Table 2) decreases in the order of Pd/LSM-3 > Pd/LSM-2 > Pd/LSM-1. The results indicate that the Pd/LSM-x samples possess much larger amount of oxygen vacancy. It is in consistence with the results observed in our previous studies of the H_2_-TPR and O_2_-TPD.

### Catalytic combustion of methane

**Figure 6:**
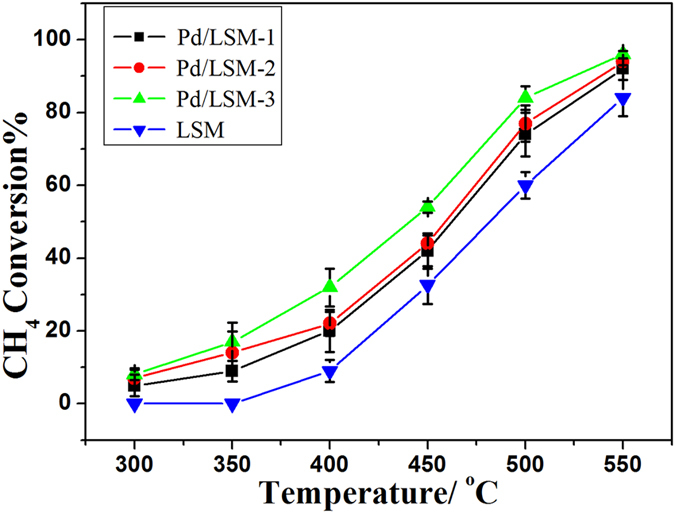
conversion of CH_4_ over LSM and Pd/LSM-x (X = 1, 2 and 3) catalysts.

The catalytic performances of the LSM and Pd/LSM-x catalysts were investigated as a function of reaction temperature in the range of 300–550 °C for methane combustion and presented in Fig. 6 and Figure S3. It is worth pointing out that CH_4_ was completely oxidized to CO_2_ and H_2_O over all the samples, and no other incomplete oxidation products such as CO were detected in the catalytic system. The results showed that each catalyst displayed an S-shaped activity profile. The methane conversion increased with the temperature, and the Pd/LSM-x catalysts performed much better than the LSM catalyst. It is convenient to compare the catalytic activities of the samples by using the reaction temperature at the methane conversion of 10%, 50% and 90% that are donated as T_10%_, T_50%_ and T_90%_ (Table S2). It is found that the catalytic performance decreased in the sequence Pd/LSM-3 > Pd/LSM-2 > Pd/LSM-1 > LSM. Obviously, the Pd/LSM-3 catalyst performed the best activity among these catalysts, giving T_10%_ and T_50%_ of 314 and 440 °C, respectively, which were much lower by 84 and 45 °C than others (Table S2). The activities were also measured over the Pd/LSM with 5.1% Pd amount (denoted Pd/LSM-4) displayed in Figure S4. Compared with the Pd/LSM-3 with 4.2% Pd content, it can be seen that the methane conversion was not significantly increased with the increase in the Pd content from 4.2% to 5.1%. Thus, the Pd/LSM catalyst with much higher Pd amount has been investigated in this study.

**Figure 7:**
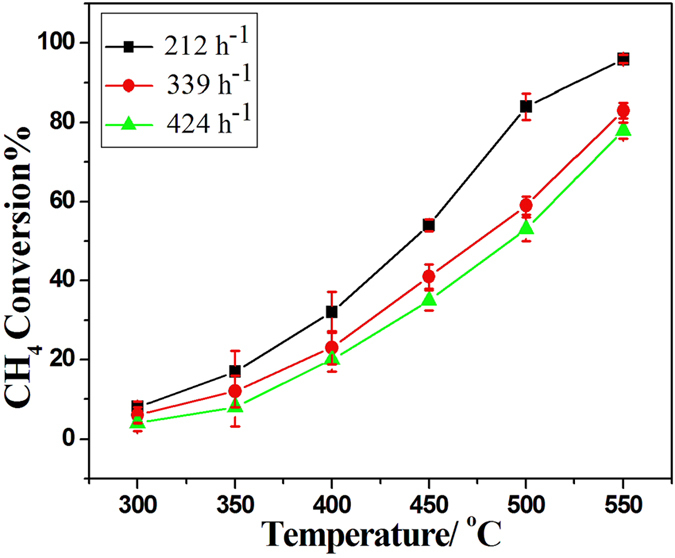
Conversion of CH_4_ over Pd/LSM-3 catalyst under different GHSV.

**Figure 8:**
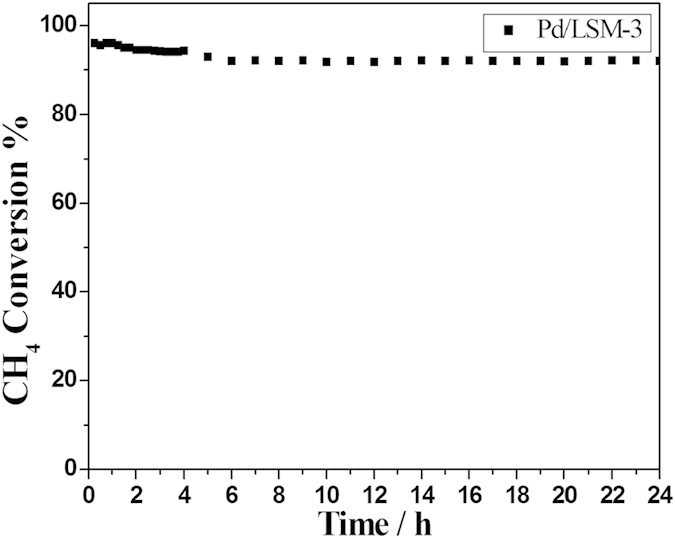
Conversion of CH_4_ with reaction time over the Pd/LSM-3 catalyst at 550 °C.

The conversion of CH_4_ over the Pd/LSM-3 catalyst under different space velocity was also tested and shown in Fig. 7. It is clear that the increase of space velocity only resulted in a certain extent decrease in the activity, but the catalysts could also show over 80% conversion of CH_4_ at 550 °C under a rather high space velocity of 424 h^−1^, suggesting that Pd/LSM-x catalyst can be highly resistant to large space velocity. The long-term reaction stability of Pd/LSM-3 catalyst was investigated at 550 °C presented in Fig. 8. The slight differences for the conversions of CH_4_ can be clearly observed with increasing time on stream over the Pd/LSM-3 catalyst. The conversion of CH_4_ was slightly decrease and still got to about 92% after 24 h over the Pd/LSM-3 at 550 °C, which indicates that the Pd/LSM-3 catalyst is quite stable with high activity under our reaction conditions. For comparison, the activities of Pd/LSM-3 catalyst are largely higher than those reported in literatures^1^,^41^,^42^. The T_10%_ reported by Baylet *et al.* over a series of 1.0 wt% Pd/REF and Pd/HCa is found to vary between 420 and 660 °C^41^. Davide Ferri *et al.*^1^ reported T_10_% for a series of 2 wt% Pd/LaFeO_3_-x (x = 300, 500, 700, 1000 °C calcined ) is found to vary between 360 °C and 520 °C. Similarly, Fernando F.M. *et al.*^42^ reported T_50_% is higher than 500 °C for 1 wt% Pd/Gd_0.1_Ce_0.9_O_1.95_ samples prepared by Incipient wetness impregnation.

The Pd/LSM-x catalysts exhibit much higher activity than the LSM for the CH_4_ combustion reaction. In H_2_-TPR, it can be seen that the amount of adsorbed oxygen in the Pd/LSM-x catalysts is higher than that in LSM catalyst, which is a key activity species in the process of methane combustion. The O_2_-TPD results also confirm it. The XPS results also agree with the above discussion of H_2_-TPR and O_2_-TPD. As we known, the surface adsorbed oxygen species is very active for the oxidation of hydrocarbons at low temperatures^12^,^22^. A high concentration of the adsorbed oxygen species would be beneficial for enhancement in catalytic activity for the total oxidation of methane^12^,^22^. In this study, more oxygen vacancies on the surface of Pd/LSM-x catalysts can be generated. This may be the reason of the Pd/LSM-x have a better activity than the LSM sample. It is well known that the catalytic activity for the methane combustion depends strongly on the chemical state of palladium. It is commonly accepted that at low temperature the active phase is crystalline PdO, which may exist in more than one form depending on the oxidized particles size and on the nature of the support, but at high temperature metallic Pd is the active phase for methane oxidation^25^. In our study, TEM results show the presence of both PdO and Pd phases in the Pd/LSM-x catalysts. The XPS results suggested that the conversion to methane was a function of Pd^2+^/Pd^0^ (Table 2; Fig. 5A). The higher ratio of Pd^2+^/Pd^0^ exists, the better catalytic activity shows. The XPS results show that the ratio of Pd^2+^/Pd^0^ decreases in the order of Pd/LSM-3 > Pd/LSM-2 > Pd/LSM-1. So the Pd/LSM-3 exhibits the best activity than the Pd/LSM-2, Pd/LSM-1 and LSM samples.

## Conclusion

The LSM and Pd/LSM-x catalysts were prepared by a facile microwave irradiation method in few minutes. The Pd/LSM-x catalysts exhibit much higher activity than the LSM for the CH_4_ combustion reactions, meanwhile, the Pd/LSM-3 catalyst with 4.2wt%Pd shows an excellent catalytic activity and stability for methane combustion, and above 92% conversion of methane can be kept for 24 h at 550 °C. It can be attributed to the high ratio of Pd^2+^ to Pd^0^ and more surface oxygen vacancy sites, which more easily for CH_4_ adsorption and oxidation of CH_4_.

## Methods

### Preparation of Catalysts

La_0.8_Sr_0.2_MnO_3.15_ was prepared by microwave irradiation process method (MIM). Aqueous solution of nitrates were used as starting materials (Mn(NO_3_)_2_, La(NO_3_)_3_·6H_2_O, Sr(NO_3_)_2_). A typical process was as follows: lanthanum nitrate (1.4805 g), strontium nitrate (0.1809 g) and manganese nitrate (1.5296 g) were dissolved in 20 mL distilled water, under ultrasound irradiation for 30 min, and stirring for 5 h at 40 °C. The resulting solution was subjected to microwave irradiation in a simple domestic oven (LWMC-201 China) operating at 2.45 GHz with a maximum power of 650 W for 6 min.

Pd/LSM-x (x = 1, 2 and 3) catalysts were prepared as theoretical Pd loading of 2 wt.%, 4 wt.% and 6 wt.%, respectively. LSM(0.9800 g, 0.9600 g, 0.9400 g) was impregnated with the aqueous solution of PdCl_2_ (mass concentration: 1% 3.3326 g, 6.6652 g, 9.9978 g) using the incipient wetness technique. Ethylene glycol (60 mL, 120 mL, 360 mL) was reducing agent, and ammonia was precipitant. The obtained solution was stirring for 24 h at 40 °C, and the resulting solution was subjected to microwave irradiation in a operating with a maximum power of 650 W for 2 min. Chloride anions are considered as a poison for Pd/LSM catalysts, so a wash step after heat is necessary to get chloride anions eliminated. After washing and filtration, the precipitates were dried at 100 °C.

### Characterization of Catalysts

X-ray diffraction (XRD) patterns were obtained with a D/MAX-3B X-ray Diffractometer (Rigaku Co.), using Cu Kα radiation combined with a Ni-filter. BET surface area was derived from N_2_ adsorption-desorption isotherms at 77 K with a Micromeritics Tristar II surface area and a porosimetry analyzer. In each case, the catalyst (more than 100 mg) was degassed under vacuum at 150 °C for 5 h before the measurement. The content of palladium was determined by inductively coupled plasma (ICP) analysis. Transmission electron microscopy (TEM) measurements were taken on a JEM-2100 electron microscope operating at 200 kV. Temperature-programmed reduction with hydrogen (H_2_-TPR) were carried out in a full automatic instrument (XQ TP-5080, China) and performed in the following procedure: Firstly, 20 mg of the catalyst was mounted in a quartz tube and calcined under O_2_ stream (30 mL/min) at 200 °C for 1 h. After the catalyst was cooled down to 25 °C, with a 30 mL/min stream of reduction gas (mixed 5% H_2_ and 95% N_2_), the reactor was carried out by raising the temperature from 20 to 900 °C at a rate of 10 °C/min. Temperature-programmed desorption of oxygen (O_2_-TPD) were carried out in the same automatic instrument of H_2_-TPR and the procedure was performed as follows: firstly, 100 mg of the catalyst was mounted in a quartz tube and calcined under a helium stream (20 mL/min) at 200 °C for 1 h, then pure O_2_ was introduced into the system at the rate of 20 mL/min for 1 h. After the catalyst was cooled down to 25 °C, the catalyst was flushed in He flow (20 mL/min) to remove physisorbed O_2_ at 25 °C. Finally, the sample was gradually heated from 25 °C to 900 °C at a ramp of 10 °C/min. X-ray photoelectron spectroscopy (XPS) was recorded on a Thermo ESCALAB 250 spectrometer using a monochromatic AlKα X-ray source (15 KV, 150 W) and analyzer pass energy of 100 eV. Binding energies (BE) are referenced to the C (1 s) binding energy of carbon taken to be 284.7 eV.

### Catalytic evaluation

CH_4_ oxidation was carried out with a fixed-bed reactor with a 6 mm-diameter glass tube, 0.100 g of the catalyst (40–60 mesh) was set in the reactor by using quartz wool, gaseous mixtures of CH_4_/O_2_/N_2_ = 1/4/95 were fed to the catalyst bed after being blended at a rate of 25 mL/min. The gas composition was analyzed before and after the reaction by an online gas chromatography using TDX-01 column (2 m × 3 mm). The activity of CH_4_ oxidation reaction was evaluated by the following equation:

CH_4_ conversion% = {[CH_4_]_in_ – [CH_4_]_out_}/[CH_4_]_in_ × 100%.

## Additional Information

**How to cite this article**: Wang, W. *et al.* Preparation of Pd supported on La(Sr)-Mn-O Perovskite by microwave Irradiation Method and Its Catalytic Performances for the Methane Combustion. *Sci. Rep.* **6**, 19511; doi: 10.1038/srep19511 (2016).

## Supplementary Material

Supplementary Information

## Figures and Tables

**Table 1:** Physico-chemical properties of the catalysts.

Catalyst	BET surface area (m^2^·g^−1^)	Pd content^a^ wt%	Date of H_2_-TPR (mmol/g)					
			T_1_^b^ (^o^C)	A_1_^c^ (mmol/g)	T_2_^b^ (^o^C)	A_2_^d^ (mmol/g)	T_3_^b^ (^o^C)	A_3_^d^ (mmol/g)
LSM	15 ± 0.6	–	–	–	326/426	0.36/2.04	782	0.47
Pd/LSM-1	19 ± 1.1	1.2	55	7.4 × 10^−3^	309	0.43	765	0.47
Pd/LSM-2	21 ± 0.8	2.6	59	3.7 × 10^−2^	305	0.46	766	0.47
Pd/LSM-3	41 ± 1.2	4.2	59	4.3 × 10^−2^	261	0.50	766	0.47

^a^Chemical compositions determined by the ICP technique.

^b^The peaks position in H_2_-TPR.

^c^Released H_2_ based on quantitative analysis of H_2_-TPR profiles.

^d^The data of H_2_ consumption in H_2_-TPR profiles.

**Table 2:** XPS data of the LSM and Pd/LSM-x catalysts.

Catalyst	Pd 3d_5/2_			Mn 2p_3/2_			O _1s_		
	Pd^0^	Pd^2+^	Pd^2+^/Pd^0^	Mn^3+^	Mn^4+^	Mn^4+^/Mn^3+^	O_lat_	O_ads_	O_ads_/O_lat_
LSM	–	–	–	641.3	642.5	1.6	529.1	531.6	1.9
Pd/LSM-1	334.9	335.6	1.0	641.0	642.3	1.1	528.4	530.6	11.7
Pd/LSM-2	334.8	335.6	1.3	460.9	642.3	0.91	529.0	531.5	12.5
Pd/LSM-3	334.4	335.2	1.4	640.2	641.5	0.87	528.7	531.6	12.7
