# Supplementary material for: Preparation of Pd supported on La(Sr)-Mn-O Perovskite by microwave Irradiation Method and Its Catalytic Performances for the Methane Combustion
Source: Sci Rep. 2016 Jan 19;6:19511. doi: 10.1038/srep19511 (PMC4726093; doi:10.1038/srep19511)
Supplement: Supplementary Information [file srep19511-s1.pdf]

Supplementary Information

Preparation of Pd supported on La(Sr)-Mn-O Perovskite by microwave Irradiation Method and Its Catalytic Performances for the Methane Combustion

Wei Wang, Fulong Yuan, Xiaoyu Niu*, Yujun Zhu*

Key Laboratory of Functional Inorganic Material Chemistry (Heilongjiang University), Ministry of Education, School of Chemistry and Materials, Heilongjiang University, Harbin, 150080 P. R. China

Table S1 2ϑ values of the diffraction peaks for the LSM and Pd/LSM-X catalysts

Catalysts	2ϑ values (°)							
LSM	23.07	32.85	40.35	46.97	52.72	58.20	68.64	77.81
Pd/LSM-1	22.93	32.58	40.13	46.75	52.32	58.07	68.11	77.67
Pd/LSM-2	22.67	32.51	40.11	46.62	52.32	57.94	68.11	77.67
Pd/LSM-3	22.67	32.49	40.00	46.71	52.32	57.92	67.98	77.63

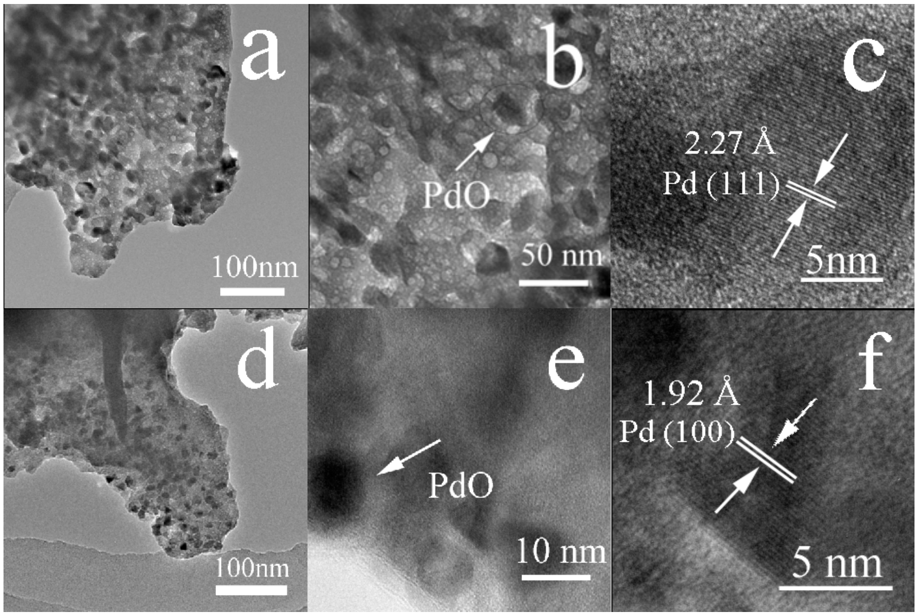

Figure S1 TEM images at increasing magnifications of Pd/LSM-1 (a-c) and Pd/LSM-3(d-f) samples.

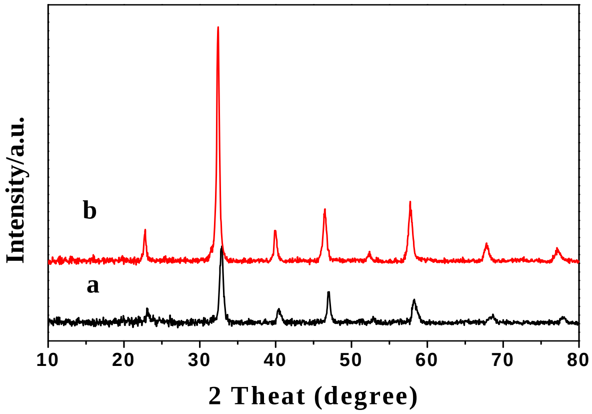

Figure S2.XRD patterns of LSM before and after reduction: (a) before reduction and (b) after reduction at 500 ^{o}C under a 10% H_2/N_2 atmosphere

Table S2 The temperature of catalysts at $T_{10\%}$, $T_{50\%}$ and $T_{90\%}$

Catalysts	$T_{10\%}$ (°C)	$T_{50\%}$ (°C)	$T_{90\%}$ (°C)
LSM	405	485	>550
Pd/LSM-1	355	460	544
Pd/LSM-2	326	455	537
Pd/LSM-3	314	440	520
Pd/LSM-3(40mL)	329	474	>550
Pd/LSM-3(50mL)	356	490	>550

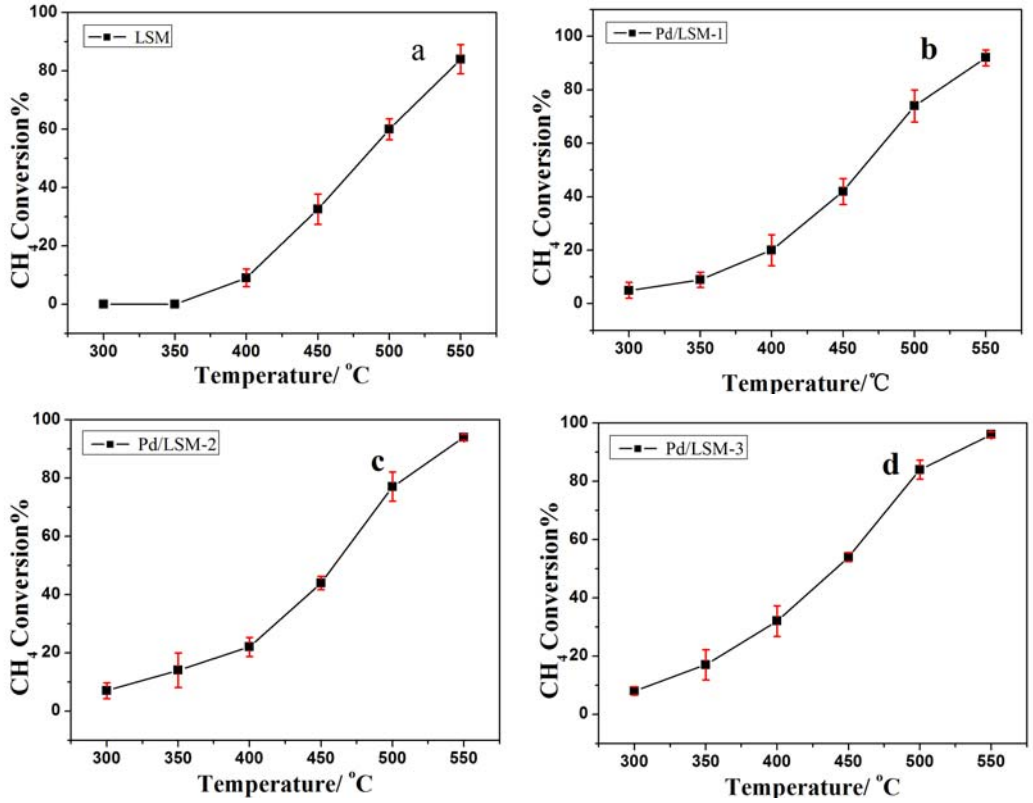

Figure S3 conversion of CH_4 over LSM and Pd/LSM-X (X=1, 2 and 3) catalysts

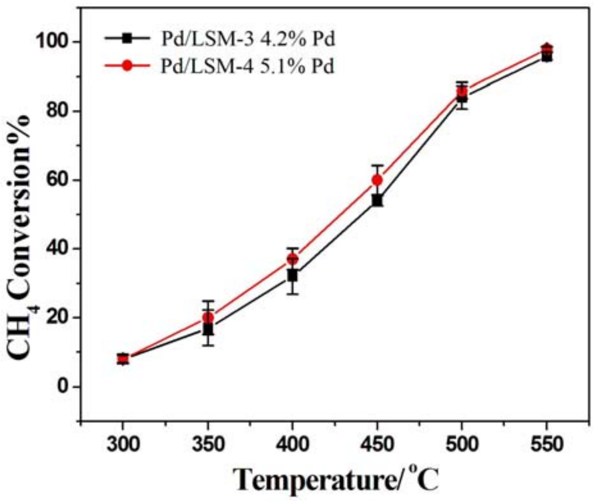

Figure S4 conversion of CH_4 with reaction temperature over the Pd/LSM-3(4.2% Pd) and Pd/LSM-4 (5.1% Pd)
